# Association between cardiometabolic index and gallstones: a cross-sectional study based on NHANES 2017–2020

**DOI:** 10.1186/s40001-025-03446-x

**Published:** 2025-11-24

**Authors:** Zhongqiao Lu, Deshan Zong, Zhongde Yang, Yingxia Hu, Bin Yue

**Affiliations:** 1 Department of Cardiovascular Diseases, The People’s Hospital of Wenshan Prefecture, Wenshan, 663000 Yunnan China; 2 Department of Gastroenterology, The People’s Hospital of Wenshan Prefecture, Wenshan, 663000 Yunnan China; 3 Department of Gastroenterology, The People’s Hospital of Wenshan Prefecture; Affiliated Wenshan Hospital, Kunming University of Science and Technology, Wenshan, 663000 Yunnan China

**Keywords:** Gallstones, Cardiometabolic index, Cross-sectional study, NHANES, Nomogram

## Abstract

**Background:**

Gallstone formation is strongly influenced by obesity, and timely detection is generally associated with improved clinical outcomes. This study aimed to investigate the relationship between cardiometabolic index (CMI) levels and gallstone occurrence.

**Methods:**

This cross-sectional study included 3743 adults. To comprehensively assess the association between the CMI and gallstone prevalence, we employed multivariable logistic regression, smoothed curve fitting, threshold effect analysis, and subgroup stratification. Key predictors of gallstones were identified using the least absolute shrinkage and selection operator (LASSO) method. A predictive nomogram incorporating the selected variables was developed, and its diagnostic performance was evaluated using the area under the receiver operating characteristic (AUROC) curve.

**Results:**

A significant association was observed between the CMI and the likelihood of gallstone occurrence. Smoothed curve fitting indicated a saturation effect in this relationship, with a threshold identified at a CMI value of 1.83. Subgroup analyses revealed consistent associations across most strata, except for gender-based stratification. The nomogram, developed using variables selected by LASSO and multivariable logistic regression, demonstrated good discriminative performance, with an area under the curve (AUC) of 0.720 (95% CI 0.695–0.746).

**Conclusion:**

Elevated CMI levels are positively associated with gallstone occurrence. The nomogram, developed using gender, age, race, hypertension, diabetes, and CMI, shows a strong association with gallstone risk and may serve as a practical tool for risk prediction.

**Supplementary Information:**

The online version contains supplementary material available at 10.1186/s40001-025-03446-x.

## Introduction

Cholelithiasis is a condition defined by solid crystalline formations in the gallbladder or biliary system, mainly consisting of cholesterol (known as cholesterol stones) or bilirubin (referred to as pigment stones) [1]. As a prevalent disease of the digestive system, cholelithiasis affects about 10–20% of adults globally, with women being twice as likely to be affected as men, and the risk rising progressively with age. Cholesterol stones comprise about 80% of cases in Western nations, whereas pigment stones are relatively more common in Asian regions [2–4]. Key risk factors include obesity, metabolic syndrome, rapid weight fluctuations, and genetic susceptibility [5, 6]. The clinical impact of cholelithiasis extends beyond typical symptoms such as biliary colic, potentially leading to acute cholecystitis, cholangitis, or biliary pancreatitis, and even life-threatening complications like gallbladder perforation and sepsis [7–9]. Consequently, timely identification and intervention for gallstones are crucial to preventing severe outcomes. As medical technology advances, the identification of more accurate novel biomarkers could enhance early detection and therapeutic strategies for gallstones.

The Cardiometabolic Index (CMI), introduced by Wakabayashi and colleagues in 2013, is an all-encompassing metabolic marker that integrates two major cardiovascular risk components: dyslipidemia (TG/HDL-C ratio) and central obesity (Waist-to-Height Ratio, WHtR). This index not only provides insight into the level of insulin resistance and the buildup of visceral fat but is also easy to compute without requiring intricate testing procedures [10–12]. Emerging research has shown that CMI is strongly linked to cardiovascular disease development and may act as an early predictor of cardiac events [13, 14]. Additionally, CMI is strongly correlated with several diseases, including diabetes, hepatic steatosis, and urinary tract stones, expanding its potential as a promising biomarker [15–17].

However, the potential association between CMI and gallstone formation remains underexplored. Therefore, this study analyzed data from the 2017–2020 National Health and Nutrition Examination Survey (NHANES) to examine this relationship and provide theoretical support for future strategies in early diagnosis and intervention.

## Methods

### Study population

**Fig. 1:**
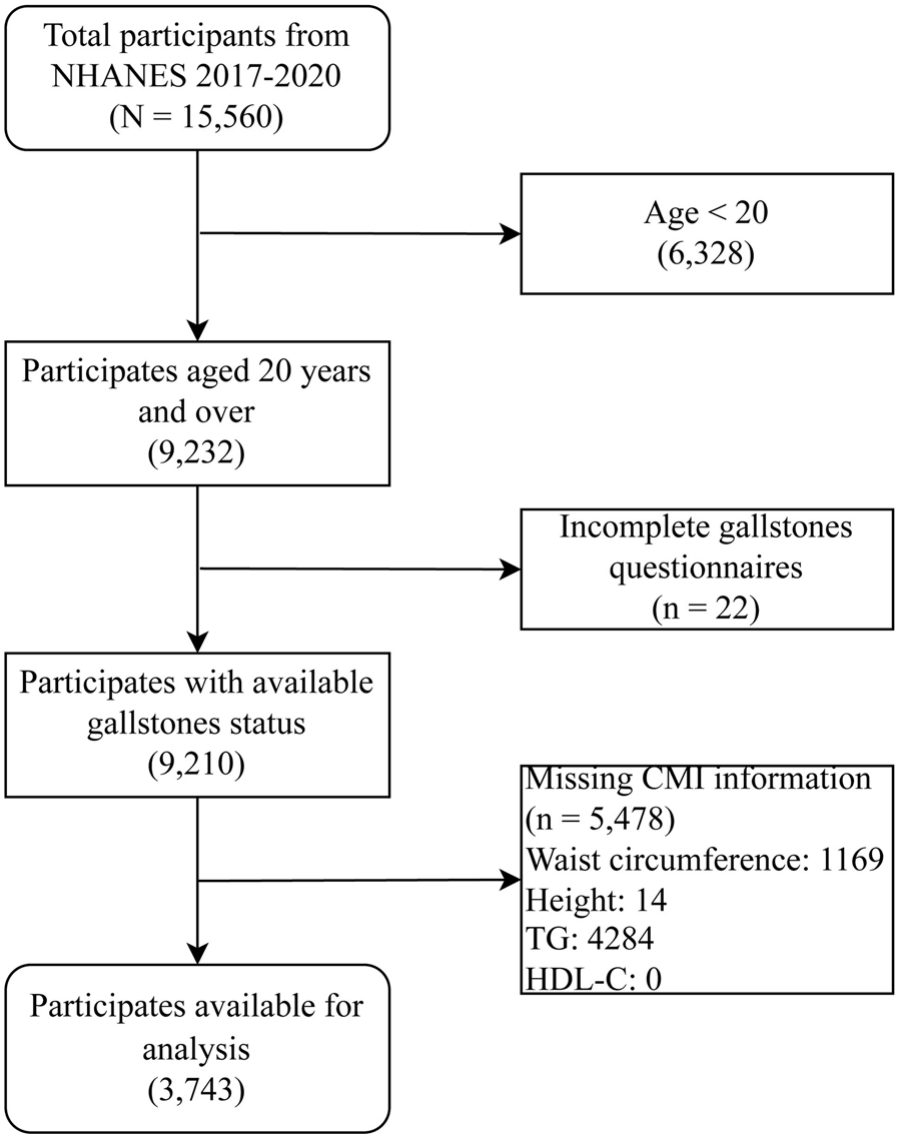
Flows of the study

NHANES, overseen by the US Centers for Disease Control and Prevention (CDC), is a cross-sectional program representing the nation. It evaluates the health and dietary status of Americans through a multistage, stratified sampling strategy, offering critical data to inform public health initiatives. This research employed NHANES data spanning from 2017 to 2020, relying on the presence of necessary exposure and outcome variables within the dataset. The flowchart of the study, shown in Fig. 1, was created based on these exclusion criteria: (1) Participants are under the age of 20; (2) Incomplete responses to the gallstone questionnaire and (3) Lack of any required variables for CMI calculation, such as height, waist circumference (WC), triglycerides (TG), or high-density lipoprotein cholesterol (HDL-C). Ultimately, 3743 participants were involved.

### Gallstone definition

Gallstone identification among participants was established through their responses to a specific inquiry: “Did you have a doctor or other health professional tell you that you have gallstones?” Individuals who responded affirmatively with a “yes” were categorized as having gallstones, thereby placing them in the gallstone group. Conversely, those who indicated “no” to the question were classified into the non-gallstone group [18].

### Exposure factors measurement

The primary focus of the research was to ascertain the cardiac metabolic index, which served as the exposure variable under investigation. This is derived from a specific formula outlined below [11]. TG and HDL-C, used to calculate the index, were measured from fasting blood samples collected after an overnight fast (at least 8 h but less than 24 h), following the NHANES laboratory protocol. Additionally, WC and other relevant high-level data were collected at a mobile screening center, ensuring a comprehensive approach to gathering the necessary information for the analysis. The units of TG and HDL-C were mg/dL, and those of WC and height were centimeters.

$$CMI =\frac{TG}{HDL-C}\times \frac{WC}{Height}$$

### Covariables

This research examined various demographic factors, including gender, age, ethnicity, educational level, marital status, and the proportion of family income relative to the poverty line. Additionally, anthropometric measurements like weight and body mass index (BMI), as well as total cholesterol levels, were gathered at the Mobile Examination Center. Questionnaire-based variables comprised lifestyle factors and comorbid conditions. Individuals who consumed at least 100 cigarettes were categorized as smokers. Participants were stratified into five categories according to their daily alcohol intake: Never drinkers (less than 12 drinks in a lifetime); Former drinkers (≥ 12 drinks in any single year and no alcohol in the past year, or no alcohol in the past year despite a lifetime consumption of ≥ 12 drinks); Mild drinkers (defined as an average daily intake of ≤ 1 drink for women and ≤ 2 drinks for men over the preceding 12 months); Moderate drinkers (averaging 1–3 drinks per day for women and 2–4 drinks for men); and Heavy drinkers (averaging ≥ 4 drinks per day for women and ≥ 5 drinks for men during the past 12 months) [19]. They also provided affirmative responses that led to the recording of self-reported diagnoses for diabetes, hypertension, and hyperlipidemia.

### Statistical analysis

CMI was categorized into four distinct quartiles (Q1–Q4). The lowest quartile, referred to as Q1, is the baseline group. Means ± standard deviations (mean ± sd) were used for continuous variables, with group differences assessed through t-tests. Categorical variables were shown as percentages [n (%)] and analyzed through chi-square tests. The evaluation of the relationship between CMI and gallstone occurrence, along with the estimation of *ORs* and their corresponding 95% *CIs*, was conducted through multivariable logistic regression analysis. Three distinct logical models were developed to analyze the data effectively. The first model, referred to as Model 1, is an unadjusted version that does not account for any external variables. This model serves as a baseline for comparison, allowing researchers to observe the raw relationships within the data without any modifications. Model 2 incorporates adjustments for several demographic factors, including gender, age, race, education level, and marital status. By including these variables, Model 2 aims to provide a more nuanced understanding of the data by controlling for the potential confounding factors under investigation. Finally, Model 3 represents a comprehensive approach, as it includes full adjustments for all relevant covariates. This model seeks to capture the most accurate relationships by considering a wide range of influencing factors, thereby enhancing the robustness of the findings. To evaluate the possible nonlinear connection between CMI and gallstones, smoothed curve fitting (SCF) was used. Piecewise linear regression was applied to examine threshold effects. Furthermore, to examine potential variations among different populations, subgroup and interaction analyses were conducted.

LASSO regression was utilized to evaluate all covariates, which encompassed the CMI indicator. The penalty parameter λ was determined via tenfold cross-validation repeated 1,000 times. Variables were selected according to the penalty corresponding to one standard error above the λ associated with the minimum prediction error (λ.1se) [20, 21]. Multivariable logistic regression analysis was then conducted to identify variables associated with the formation of gallstones. Ultimately, a nomogram was developed and subsequently assessed by the receiver operating characteristic (ROC) curve.

Covariates with less than 20% missing data were imputed using multiple imputation methods. A *P*-value below 0.05 was deemed statistically significant. All statistical evaluations were conducted utilizing R 4.3 and the Empower (R) software package (www.empowerstats.com, X&Y solutions, Inc., Boston, MA, US).

## Results

### Baseline characteristics

The final analysis comprised 3743 participants, with 1925 of them (51.43%) identifying as female. The average age of those involved in the research was 50.71 ± 17.28 years. Gallstones were present in 397 participants (10.61%), and absent in 3346 (89.39%). The median CMI was 1.06 (0.58, 1.86). Table 1 presents a detailed overview of the participants’ characteristics. In contrast to individuals without gallstones, those with a diagnosis of gallstones demonstrated a higher likelihood of exhibiting certain characteristics: an increased percentage of females, an older age group (60 years and above), a greater representation of non-Hispanic Whites, and a marital status indicating they are divorced, separated, or widowed. Furthermore, individuals with gallstones had a higher likelihood of being diagnosed with hypertension, together with increased prevalence of diabetes and hyperlipidemia, as well as elevated CMI levels. (*P* < 0.05). Among all covariates, PIR (Ratio of family income to poverty) had the highest missing rate at 13.33% (*Supplementary Figure S1*).

**Table 1:** Baseline characteristics of participants from National Health and Nutrition Examination Survey (NHANES) 2017–2020

Variables	Overall	Gallstones		*t/χ*^*2*^	*P*-value
	(n = 3,743)	No (n = 3,346)	Yes (n = 397)		
Gender, n (%)					
Male	1818 (48.57%)	1706 (50.99%)	112 (28.21%)	73.69	< 0.001
Female	1925 (51.43%)	1640 (49.01%)	285 (71.79%)		
Age (years)	50.71 ± 17.28	49.90 ± 17.33	57.38 ± 15.37	8.22	< 0.001
Age strata, n (%)					
20–40	1143 (30.54%)	1081 (32.31%)	62 (15.62%)	55.53	< 0.001
40–60	1258 (33.61%)	1118 (33.41%)	140 (35.26%)		
≥ 60	1342 (35.85%)	1147 (34.28%)	195 (49.12%)		
Race, n (%)					
Mexican American	481 (12.85%)	423 (12.64%)	58 (14.61%)	21.87	< 0.001
Other hispanic	380 (10.15%)	328 (9.80%)	52 (13.10%)		
Non-Hispanic white	1269 (33.90%)	1112 (33.23%)	157 (39.55%)		
Non-Hispanic black	945 (25.25%)	877 (26.21%)	68 (17.13%)		
Other race	668 (17.85%)	606 (18.11%)	62 (15.62%)		
Education level, n (%)					
Less than 9th grade	280 (7.48%)	254 (7.59%)	26 (6.55%)	9.62	0.047
9-11th grade	428 (11.43%)	375 (11.21%)	53 (13.35%)		
High school graduate	892 (23.83%)	793 (23.70%)	99 (24.94%)		
Some college or associates degree	1215 (32.46%)	1072 (32.04%)	143 (36.02%)		
College graduate or above	928 (24.79%)	852 (25.46%)	76 (19.14%)		
Marital status, n (%)					
Married/Living with a partner	2222 (59.36%)	1986 (59.35%)	236 (59.45%)	15.48	< 0.001
Divorced/Separated/Widowed	807 (21.56%)	698 (20.86%)	109 (27.46%)		
Never married	714 (19.08%)	662 (19.78%)	52 (13.10%)		
PIR	2.59 ± 1.51	2.60 ± 1.52	2.51 ± 1.42	1.04	0.298
PIR strata, n (%)					
< 1.3	868 (23.19%)	781 (23.34%)	87 (21.91%)	4.35	0.226
1.3–1.85	483 (12.90%)	424 (12.67%)	59 (14.86%)		
1.85–3.5	1322 (35.32%)	1171 (35.00%)	151 (38.04%)		
≥ 3.5	1070 (28.59%)	970 (28.99%)	100 (25.19%)		
Height (cm)	166.89 ± 9.96	167.26 ± 10.01	163.79 ± 9.03	6.60	< 0.001
Weight (Kg)	83.33 ± 22.29	82.63 ± 21.97	89.27 ± 24.04	5.64	< 0.001
BMI (Kg/m^2^)	29.84 ± 7.30	29.44 ± 7.04	33.22 ± 8.46	9.90	< 0.001
Waist circumference (cm)	100.76 ± 17.16	99.88 ± 16.92	108.13 ± 17.28	9.17	< 0.001
TG (mg/dL)	90.00 (60.00, 133.00)	88.00 (59.00, 131.00)	106.00 (71.00, 143.00)	2.59	0.010
TCHO (mg/dL)	184.19 ± 41.26	184.40 ± 40.91	182.37 ± 44.10	0.93	0.353
HDL-C (mg/dL)	53.71 ± 16.01	53.88 ± 16.22	52.36 ± 14.04	1.79	0.073
Alcohol use, n (%)					
Never	326 (8.71%)	294 (8.79%)	32 (8.06%)	29.93	< 0.001
Former	772 (20.63%)	651 (19.46%)	121 (30.48%)		
Mild	1360 (36.33%)	1233 (36.85%)	127 (31.99%)		
Moderate	896 (23.94%)	805 (24.06%)	91 (30.48%)		
Heavy	389 (10.39%)	363 (10.85%)	26 (8.06%)		
Smoking, n (%)					
Yes	1609 (42.99%)	1429 (42.71%)	180 (45.34%)	1.00	0.317
No	2134 (57.01%)	1917 (57.29%)	217 (54.66%)		
Hypertension, n (%)					
Yes	1441 (38.50%)	1225 (36.61%)	216 (54.41%)	47.48	< 0.001
No	2302 (61.50%)	2121 (63.39%)	181 (45.59%)		
Diabetes, n (%)					
Yes	619 (16.54%)	512 (15.30%)	107 (26.95%)	34.90	< 0.001
No	3124 (83.46%)	2834 (84.70%)	290 (73.05%)		
Hyperlipidemia, n (%)					
Yes	1376 (36.76%)	1184 (35.39%)	192 (48.36%)	25.71	< 0.001
No	2367 (63.24%)	2162 (64.61%)	205 (51.64%)		
CMI	1.06 (0.58, 1.86)	1.02 (0.56, 1.81)	1.37 (0.78, 2.19)	2.63	0.009
CMI strata, n (%)					
Q1 (< 1.576)	935(24.98%)	882(26.36%)	53(13.35%)	32.31	< 0.001
Q2 (1.576–2.099)	935(24.98%)	824(24.63%)	111(27.96%)		
Q3 (2.099–2.760)	937(25.03%)	819(24.48%)	118(29.72%)		
Q4 (≥ 2.760)	936(25.01%)	821(24.54%)	115(28.97%)		

*PIR* Ratio of family income to poverty, *BMI* Body mass index, *TG* Triglyceride, *TCHO* Total cholesterol, *HDL-C* High-density lipoprotein, *CMI* Cardiometabolic index

### Association between gallstones and CMI

Three distinct multivariate logistic regression models were developed (Table 2). A notable positive correlation was found between CMI and the presence of gallstones in Model 1. In the fully adjusted Model 3, the association between CMI and gallstones remained significant (*OR* = 1.26, 95% *CI* 1.11–1.40), indicating a 26% higher odds of gallstones per unit increase in CMI. Subsequently, CMI was categorized into quartiles for further analysis. In the fully adjusted Model 3, participants in the highest CMI quartile (Q4) had 2.10-fold higher odds of gallstones compared with those in Q1 (*OR* = 2.10, 95% *CI* 1.47–3.00), and the difference was statistically significant (*P* < 0.01).

**Table 2:** Association Between CMI and Gallstones

	Model 1 OR (95% CI)	Model 2 OR (95% CI)	Model 3 OR (95% CI)
CMI			
Continuous	1.26 (1.14, 1.40)	1.30 (1.16, 1.45)	1.26 (1.11, 1.40)
Categories			
Q1 (< 1.576)	Reference	Reference	Reference
Q2 (1.576–2.099)	2.24 (1.59, 3.15)	1.96 (1.38, 2.79)	1.88 (1.32, 2.68)
Q3 (2.099–2.760)	2.40 (1.71, 3.36)	2.21 (1.56, 3.13)	2.08 (1.46, 2.95)
Q4 (≥ 2.760)	2.33 (1.66, 3.27)	3.34 (1.65, 3.32)	2.10 (1.47, 3.00)
P for trend	< 0.001	< 0.001	< 0.001

*CMI* Cardiometabolic index

Model 1: no covariates were adjusted

Model 2: Sex, age, race, education level, marital status were adjusted

Model 3: Sex, age, race, education level, marital status, PIR, alcohol use, smoking, hypertension, diabetes, and hyperlipidemia were adjusted

### SCF and threshold effect analysis

**Fig. 2:**
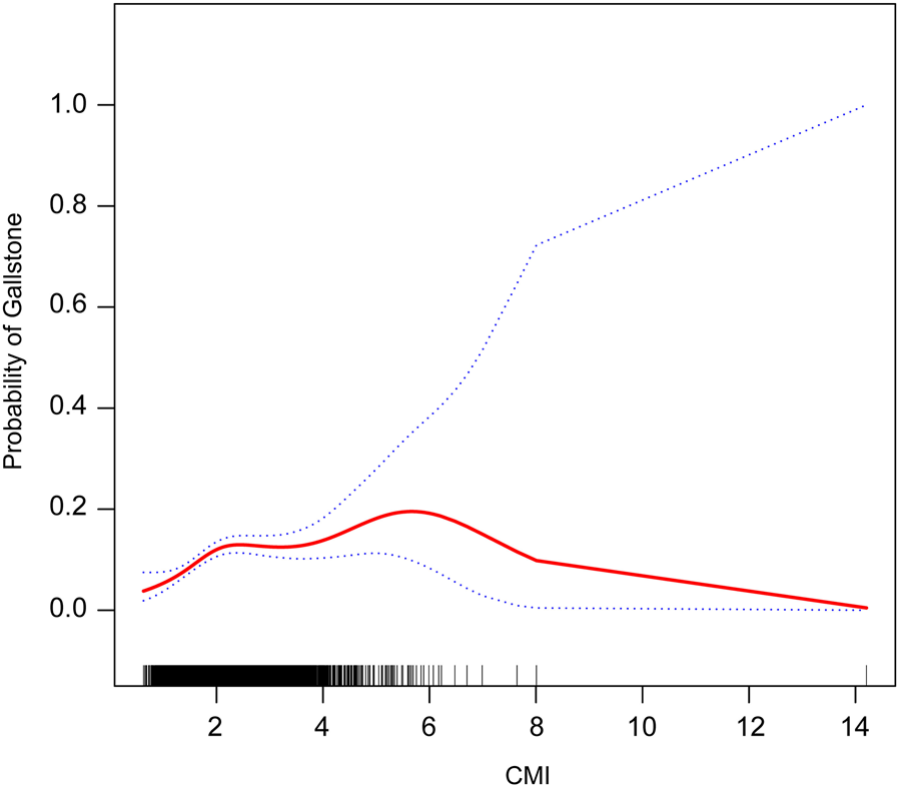
The nonlinear relationship between CMI and gallstones. The solid red line indicates a smooth curve fit between CMI and probability of gallstones. The blue band indicates the 95% confidence interval of the fit. CMI, Cardiometabolic index

To provide additional insight into the connection between CMI and gallstones, an analysis using SCF was performed according to Model 3 (Fig. 2). This analysis uncovered a nonlinear relationship between CMI and the occurrence of gallstones. Subsequently, an analysis of the threshold effect was conducted to enhance the understanding of their connection (Table 3). The inflection point for CMI was determined to be 1.83, accompanied by a log-likelihood ratio of under 0.001. This suggests that when CMI is below 1.83, each 1-unit increase in CMI is associated with a 3.53-fold higher odds of gallstones (*OR* = 3.53, 95% *CI* 1.92–6.51). However, when CMI exceeds 1.83, further increases are not significantly associated with higher odds of gallstones. Thus, above this threshold, additional increases in CMI do not materially elevate gallstone risk.

**Table 3:** Threshold effect analysis of CMI on gallstones using a two-piecewise logistic regression model in adults in the NHANES 2017–2020

Threshold effect analysis	Gallstones *OR* (95%*CI*) *P*-value
CMI	
Inflection point of CMI (K)	1.83
< K slope	3.53 (1.92, 6.51) < 0.001
> K slope	1.07 (0.92, 1.24) 0.412
Log-likelihood ratio test	< 0.001

All covariates had been adjusted

*CMI* Cardiometabolic index

### Subgroup analysis

**Fig. 3:**
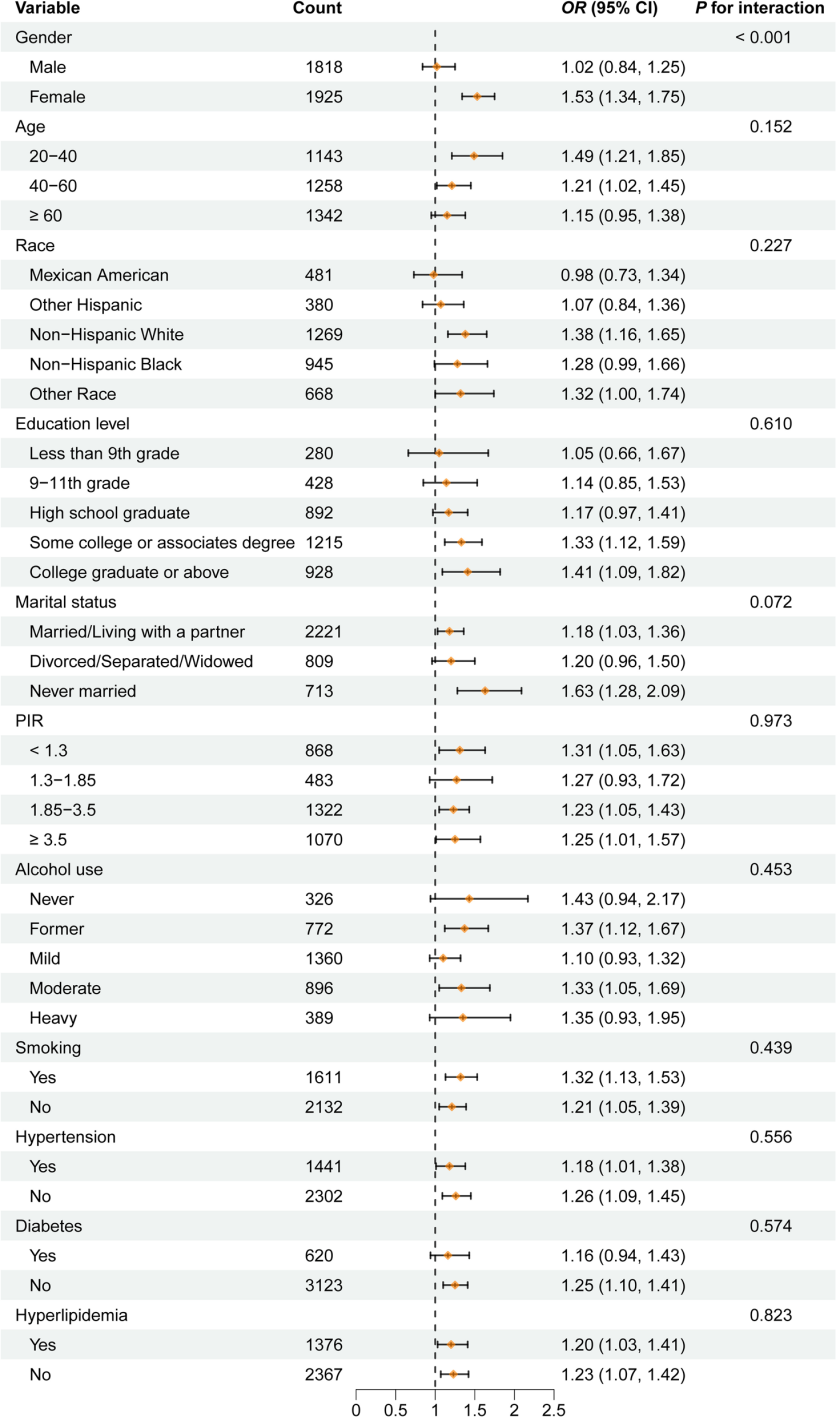
Subgroup analysis of CMI associated with gallstones. PIR, Ratio of family income to poverty

To evaluate the robustness of the association between CMI and gallstones across various subgroups, subgroup analyses were conducted. The association remained consistent in most subgroups, with no significant interactions observed (Fig. 3). It is important to note that a significant statistical interaction was observed between CMI and gender (P for interaction = 0.001), suggesting that females are at a higher risk of developing gallstones.

### Selection of variables and model construction

**Fig. 4:**
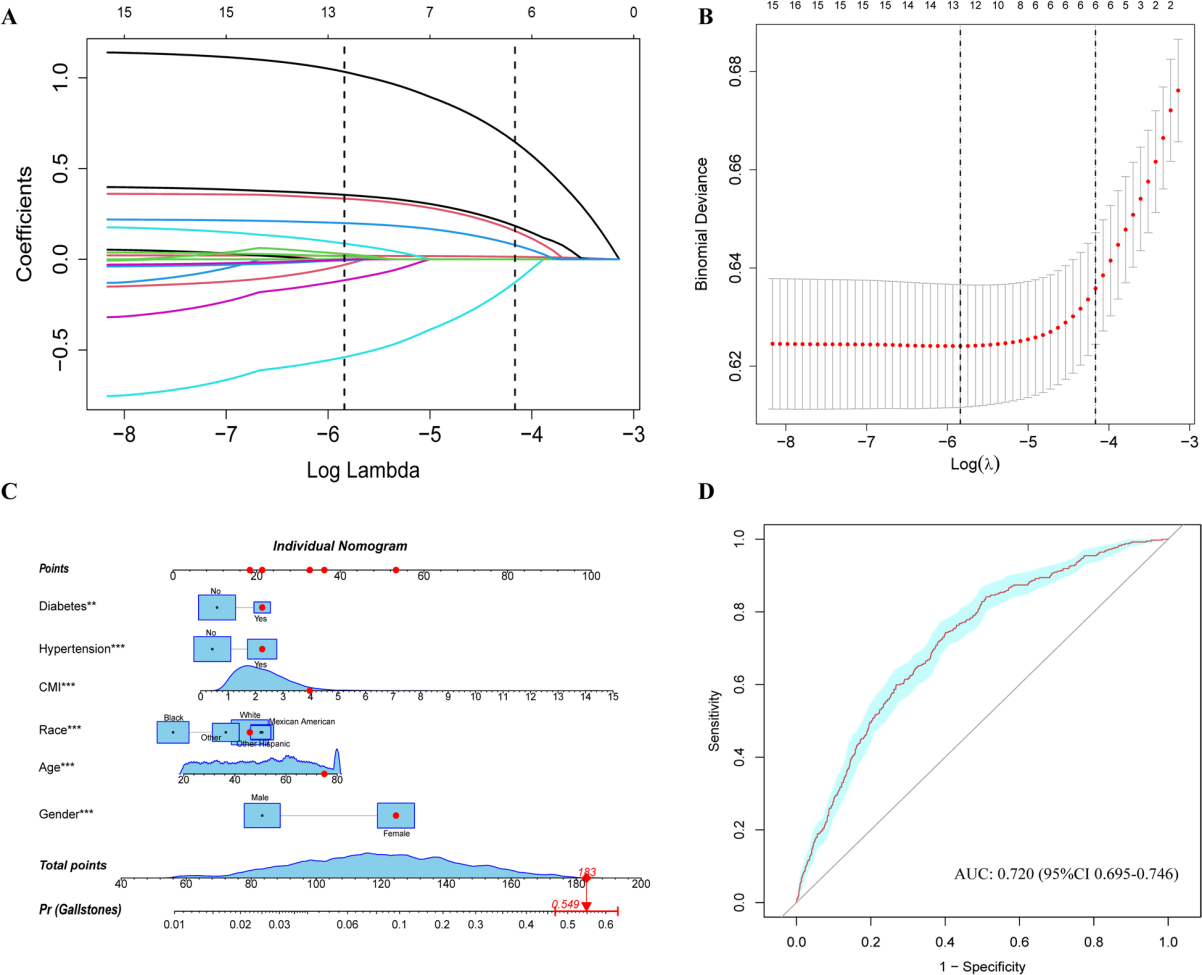
**A** Plot of LASSO regression coefficients. **B** Cross-validation curve for selecting the optimal tuning parameter (λ). **C** Nomogram model based on the key factors identified by LASSO regression; red dots represent an example for a 72-year-old female participant with gallstones. **D** ROC curve evaluating the diagnostic performance of the nomogram model

**Fig. 5:**
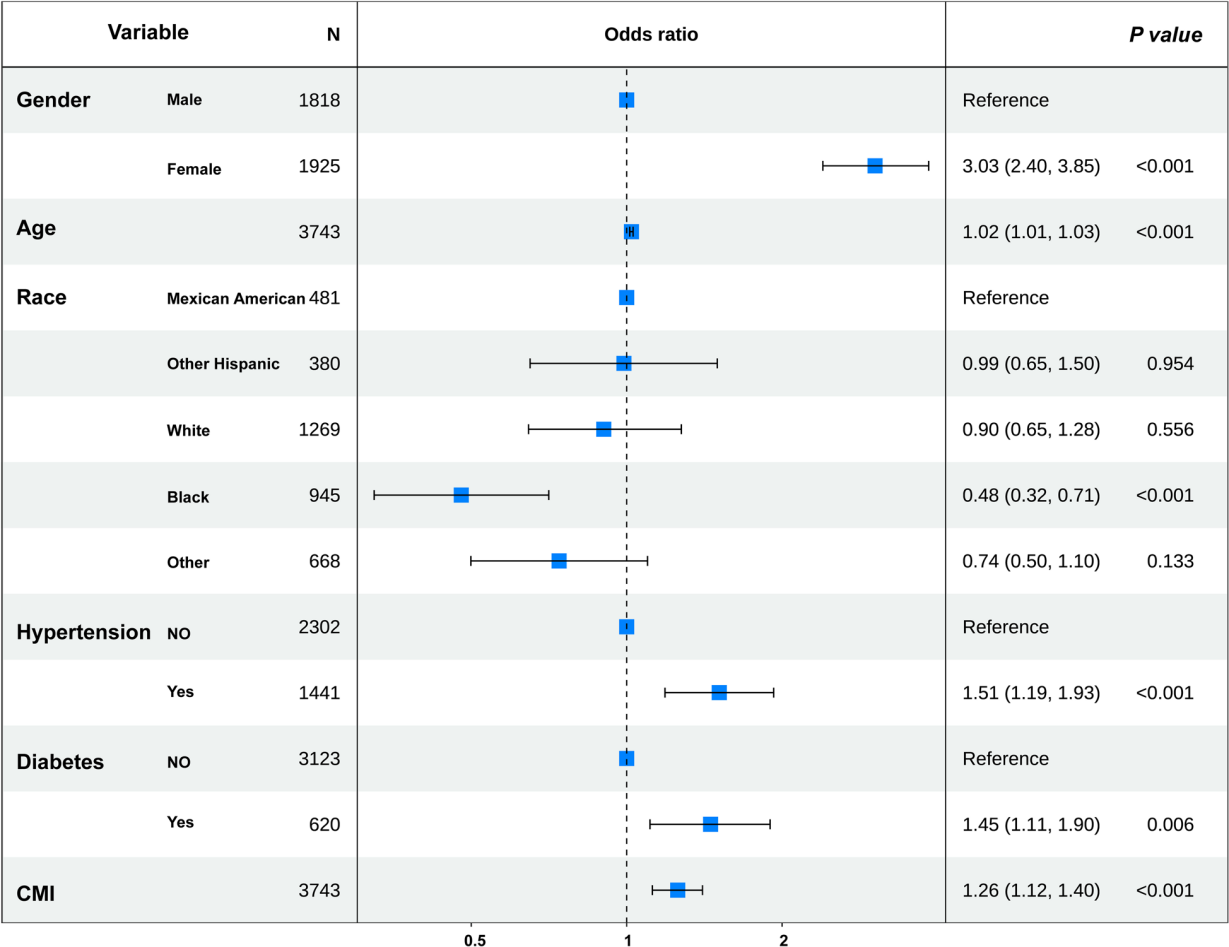
Multivariate logistic regression analysis. CMI, Cardiometabolic index

Correlation analysis was employed to preliminarily explore the relationship among continuous variables, revealing significant correlations, such as weight and WC (*Supplementary Figure S2*). To identify the key factors associated with gallstone occurrence, LASSO (Fig. 4A, B) and multivariable logistic regression analyses (Fig. 5) were employed to select variables. Subsequently, the VIF for the selected six variables, including gender, age, race, hypertension, diabetes, and CMI, was calculated to eliminate multicollinearity further (*Supplementary Figure S3*). A nomogram was developed to estimate the probability of gallstone formation (Fig. 4 C). The discriminative performance of the nomogram was further validated through ROC curve analysis (Fig. 4D). The results indicated good discriminative power of the prediction model with an AUC 0.720 (95%*CI* 0.695–0.746).

## Discussion

This study investigates the relationship between CMI and gallstones using data from 3743 eligible participants in the NHANES database. The results indicated a notably meaningful positive association between CMI and the presence of gallstones. Notably, a nonlinear connection between CMI and gallstone formation was discovered, which included a particular inflection point on the curve. These findings indicate that CMI values within a specific range are strongly associated with gallstones, implying that maintaining an optimal CMI level may have clinical significance in reducing gallstone prevalence. Additionally, the nomogram constructed based on the screened variables exhibited adequate discriminative power for detecting gallstones. These results offer important implications regarding the involvement of lipid metabolism in gallstone pathogenesis.

As major manifestations of metabolic dysfunction, obesity and lipid abnormalities are strongly associated with gallstone risk. CMI serves as a novel metric that concurrently measures the buildup of visceral fat and the imbalance of lipids. At present, studies specifically examining the role of CMI remain scarce. A cohort study conducted in Norway, which included 63,249 individuals, found that being overweight and obese increased the likelihood of undergoing cholecystectomy due to gallstone disease by 58% and 200%, respectively [22]. Likewise, markers reflecting increased visceral adiposity, such as waist circumference, show a strong positive association with gallstones [23–25], though none of these studies addressed composite metrics like CMI. Regarding lipid profiles, research conducted by Chen and colleagues identified a linear inverse relationship between HDL-C and gallstone risk.

In contrast, TG levels showed a U-shaped relationship with gallstone incidence [26]. Conversely, the findings of this research indicated a threshold effect occurring at a CMI value of 1.83, after which the risk of developing gallstones stabilized. This discrepancy may be attributed to the composite nature of CMI, combining indicators of lipid metabolism and adiposity. In addition, most previous studies relied on traditional anthropometric measures. A Japanese cohort study involving 717 participants measured subcutaneous and visceral fat using CT and ultrasound, and found that visceral fat was a better predictor of gallstone risk than BMI [27]. The finding held even among individuals with normal body weight. It is consistent with the mechanism by which CMI reflects visceral adiposity. However, CMI is more cost-effective than imaging-based assessments. It is noteworthy that other studies have shown associations between various obesity and inflammatory markers and gallstones [28–32]. For instance, an extensive prospective cohort study (n = 15,364) reported a linear association between the triglyceride-glucose index and gallstone risk [33]. Another research indicated that the metabolic scores for visceral fat exhibited a nonlinear correlation with gallstones, identifying an inflection point at 8.57 [18].

This research discovered a nonlinear relationship linking CMI to the likelihood of developing gallstones. CMI elevation below 1.83 significantly raised the risk, while further increases beyond this threshold had a diminished or non-significant impact. Multiple underlying mechanisms could explain this observation. To begin with, an increase in CMI is closely associated with insulin resistance, a condition recognized for promoting the development of cholesterol gallstones. On one hand, insulin resistance may increase biliary cholesterol secretion by upregulating hepatic cholesterol transporters ABCG5/8 [34]. On the other hand, it may suppress bile acid synthesis enzymes (e.g., CYP7B1), reducing bile acid levels, resulting in excess cholesterol in the bile and encouraging the development of gallstones [35]. Secondly, insulin resistance can impair gallbladder motility and reduce bile expulsion, leading to bile stasis and subsequent stone formation [36, 37]. Dysregulation in adipose tissue secretory function may be a key mediating factor. Excess visceral fat can secrete higher levels of hormones such as leptin and resistin, disrupting insulin signaling, aggravating insulin resistance, and impairing cholesterol metabolism and gallbladder function [38, 39]. Additionally, gut microbiota dysbiosis is intimately associated with metabolic disorders. Under conditions of excess visceral adiposity and insulin resistance, gut microbial diversity is reduced and bile acid metabolism is disrupted, which further contributes to abnormal cholesterol handling and gallstone risk [40, 41]. Lastly, inflammatory mediators released by adipose tissue, such as TNF-α and IL-6, can intensify metabolic dysregulation and abnormal cholesterol metabolism, facilitating gallstone development. These mechanisms might account for the fast-rising gallstone risk at low CMI levels due to progressive metabolic dysregulation, with a plateauing of risk beyond the threshold as metabolic effects reach saturation.

The association between CMI and gallstones was stronger among women in sex-stratified analyses. This aligns with previous studies, wherein estrogens increase hepatic cholesterol secretion and perturb bile acid metabolism, resulting in cholesterol supersaturation in bile, while progestogens reduce gallbladder contractility and prolong bile retention [42, 43]. Moreover, pregnancy, oral contraceptives, postmenopausal hormone therapy, and the postmenopausal tendency toward central fat accumulation may partly account for this observation. We further observed correlations between gallstone risk and educational attainment and marital status; however, significance was lost in stratified analyses, possibly due to limitations in our categorization scheme. Educational attainment‒central to socioeconomic status‒typically reflects healthier attitudes and behaviors [44], and it can influence dietary quality through its effects on occupation and income [45]. Marital status may confer health advantages via resource pooling, social support, health monitoring, and healthier behaviors, with stronger effects reported among men. Unfavorable marital statuses (e.g., divorce, widowhood) may precipitate unhealthy lifestyle changes and psychological stress, raising CMI and thereby increasing the risk of cholesterol supersaturation and gallstone formation [46, 47]. However, these relationships may involve substantial sex interactions that require further investigation.

Subgroup analysis also illustrated that the influence of elevated CMI on gallstone risk was significant in the 20–40 age group. Several mechanisms might underlie this finding: First, the metabolism in younger individuals (especially those aged 20–40) is more active, and visceral fat accumulation and dyslipidemia at this stage more readily lead to metabolic disorders, including insulin resistance, lipid disorders, and cholesterol metabolism abnormalities. These factors significantly contribute to the development of gallstones [48]. Second, inflammatory responses might have a more crucial influence on gallstone formation in this age group. Among young individuals, elevated CMI tends to trigger systemic inflammation, which contributes to cholesterol crystallization, gallbladder dysfunction, and disordered bile metabolism, ultimately fostering gallstone formation [18, 49]. Furthermore, in contrast to older adults, younger individuals often present with hormonal conditions (e.g., elevated estrogen) and lifestyle habits (e.g., calorie-dense diets, sedentary behavior, late-night activities) that enhance adiposity and metabolic syndrome risk, reinforcing the association between CMI and gallstones [50, 51]. Finally, younger populations may have heightened genetic and environmental vulnerability to gallstone formation, and shifts in metabolic indicators like CMI might more accurately represent their metabolic status. Therefore, as a combined indicator of visceral adiposity and lipid abnormalities, CMI shows enhanced predictive power and impact among individuals aged 20–40 [32].

As a simple, low-cost, and reproducible metabolic indicator, CMI shows promise for stratifying risk of stone formation in primary care and health screening settings. Future studies should validate the predictive value of CMI for incident stones in prospective cohorts, determine sex- and age-specific threshold values, and assess its incremental value beyond traditional indicators such as BMI, WC, and TG/HDL-C. Randomized or real-world studies are also warranted to evaluate whether CMI-guided lifestyle and metabolic management in high-risk subgroups (e.g., metabolically unhealthy women) can reduce the incidence of symptomatic stones, cholecystitis, or pancreatitis, and the need for surgical interventions.

## Study strengths and limitations

Several notable strengths characterize this analysis. First, we conducted stepwise adjustments for potential confounding factors to validate the association between CMI and gallstones. In addition, we employed SCF and threshold effect analyses to explore the nonlinear relationship between CMI and gallstones, as well as to determine the inflection point. LASSO and multivariable logistic regression were used to identify key features associated with gallstones and to construct a nomogram model with strong discriminatory power. Nonetheless, given the cross-sectional design of this study, causal inference between CMI and gallstones is limited. As a composite indicator, CMI struggles to precisely distinguish the independent effects of dyslipidemia (TG/HDL-C) and central obesity (WHtR) on gallstone risk within the final model. Moreover, since the NHANES data are based on self-reported questionnaires, the diagnosis of gallstones relied on participants’ self-reports after being informed by healthcare providers, without confirmation through imaging studies. Therefore, the results should be interpreted with caution, as recall bias may be unavoidable. To further verify the robustness and external validity of our findings, future multicenter prospective studies integrating CT- or MRI-based visceral fat quantification with clinical data are warranted to comprehensively elucidate the association among visceral fat, metabolic dysregulation, and gallstones.

## Conclusion

We demonstrated a clear and significant association between CMI levels and the development of gallstones. The predictive nomogram showed promising performance in identifying at-risk individuals. It may serve as an effective tool for early risk assessment, particularly by targeting modifiable risk factors such as CMI. Compared with imaging-based methods, this model provides a more accessible and optimized screening strategy for gallstones. To further validate these results, larger-scale cohort studies are warranted.

## Supplementary Information

Supplementary material 1.

## Data Availability

Supplementary material provides the raw data analyzed in the article. The data from this study are also publicly available on the NHANES website (https://www.cdc.gov/nchs/nhanes/index.html).
