# Supplementary material for: Association between cardiometabolic index and gallstones: a cross-sectional study based on NHANES 2017–2020
Source: Eur J Med Res. 2025 Nov 24;30:1160. doi: 10.1186/s40001-025-03446-x (PMC12642245; doi:10.1186/s40001-025-03446-x)
Supplement: Supplementary file 1 — Supplementary material 1. [file 40001_2025_3446_MOESM1_ESM.docx]

**Supplementary materials**

Figure S1: Missing values in variables

Figure S2: Heatmap of Correlations among Continuous Variables

Figure S3: Variance Inflation Factor (VIF) for Selected Variables


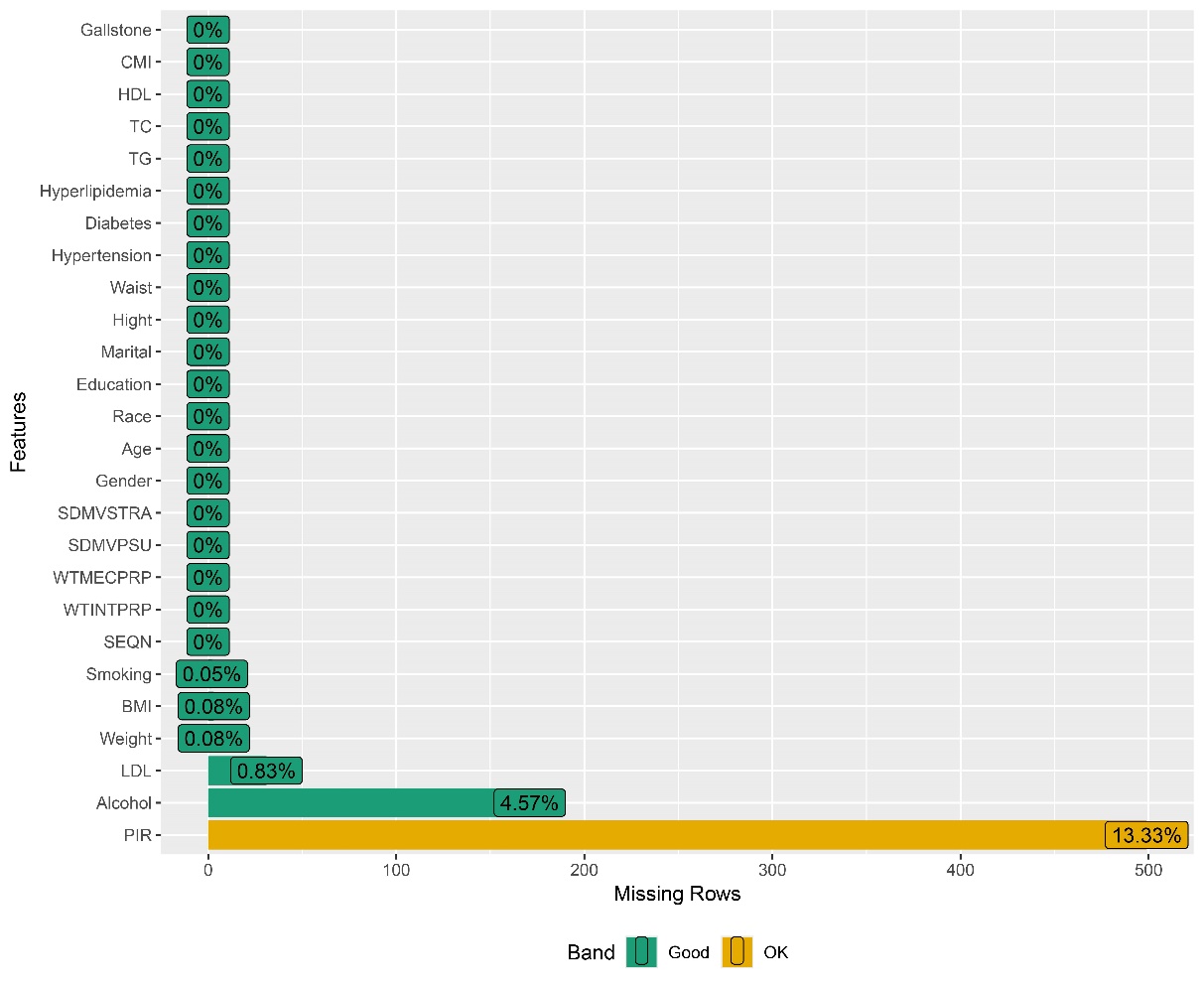


Figure S1: Missing values in variables


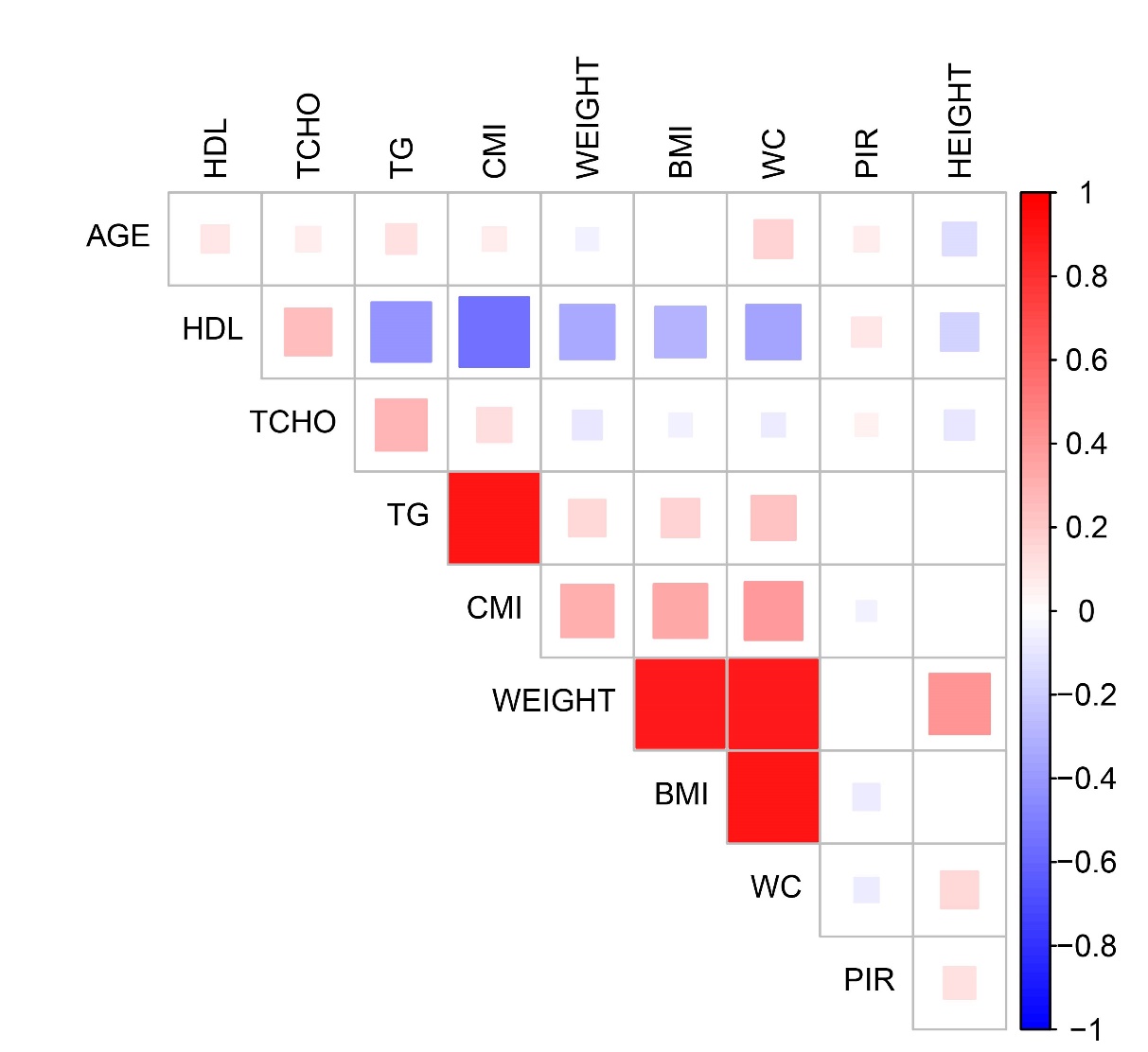


Figure S2: Heatmap of Correlations among Continuous Variables


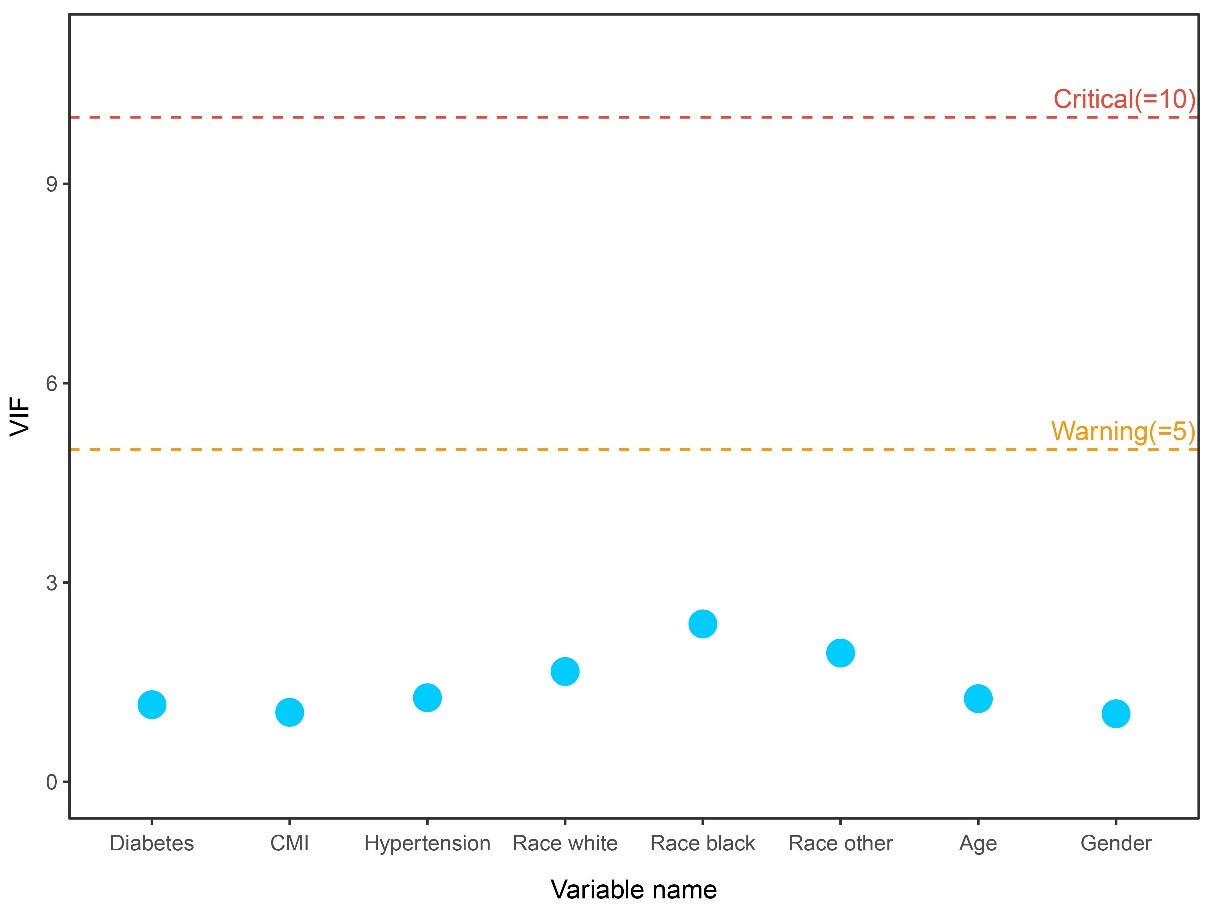


Figure S3 Variance Inflation Factor (VIF) for Selected Variables
